# A low-carb diet increases fecal short-chain fatty acids in feces of obese women following a weight-loss program: randomized feeding trial

**DOI:** 10.1038/s41598-023-45054-x

**Published:** 2023-10-24

**Authors:** Zahra Abbaspour Rad, Seyedeh Neda Mousavi, Hossein Chiti

**Affiliations:** 1 Metabolic Diseases Research Center, Zanjan University of Medical Sciences, Zanjan, Iran; 2 Department of Nutrition, School of Medicine, Zanjan University of Medical Sciences, Zanjan, Iran

**Keywords:** Obesity, Gastroenterology

## Abstract

To compare fecal level of short-chain fatty acid (SCFA) and some serum inflammatory markers between the low-carbohydrate (LCD) and the habitual (HD) diet, subjects were enrolled from our previous study on the effect of LCD *vs*. HD on gut microbiota in obese women following an energy-restricted diet. Serum interleukin-6 (IL-6) significantly increased in the HD group (*p* < 0.001). Adjusted for the baseline parameters, fecal level of butyric, propionic, and acetic acid were significantly different between the LCD and HD groups (*p* < 0.001, *p* = 0.02, and *p* < 0.001, respectively). Increase in serum insulin level correlated with decrease in fecal propionic acid by 5.3-folds (95% CI =  − 2.7,  − 0.15, *p* = 0.04). Increase in serum high sensitive C-reactive protein (hs-CRP) correlated with decrease in the percentage of fecal butyric acid by 25% (*p* = 0.04). Serum fasting blood sugar (FBS) and insulin showed a significant effect on fecal acetic acid (*p* = 0.009 and *p* = 0.01, respectively). Elevated serum FBS and insulin correlated with increase in fecal acetic acid by 2.8 and 8.9-folds (95%CI = 0.34, 1.9 and 1.2, 9.2), respectively. The LCD increased fecal SCFAs and a significant correlation was seen between serum IL-6 and fecal propionic acid level. More studies are needed to reach a concise correlation.

*Trial registration number*: The trial was registered in Iranian ClinicalTrials.gov IRCT20200929048876N3.

Due to the high prevalence and incidence of obesity and its strong relationship with all chronic complications, successful and harmless anti-obesity strategies are the primary proceeding in the health system^1^. The effect of diet on insulin sensitivity is definite^2^; however, the optimal diet is not clear. People consume macronutrients in different percentages^3^. Low carbohydrate diet can be varied in terms of carbohydrate content and quality^4^. Therefore there is no consensus on precise definitions and comparisons among studies^5^. Recently, prevalence of obesity has increased due to high carbohydrate and fat consumption across the world^6^. Low-carb diet is more popular for inducing rapid weight loss^7^. However, the side effects due to high intake of fat reduce the adherence to this pattern^8,9^. Changes in dietary fatty acids are suggested to prevent metabolic complications induced by a high fat diet^10^. It is reported that saturated fatty acids (SFAs) are more obesogenic than mono- and polyunsaturated fatty acids (MUFAs and PUFAs), because diet-induced thermogenesis are higher in a diet rich in MUFAs and PUFAs than SFAs^11^. Low-carb diet has been shown an improve effect on blood glucose, serum insulin, homeostasis model assessment of insulin resistance (HOMA-IR), and blood pressure in obese patients^12^.

Short-chain fatty acids (SCFAs) are the end product of undigested/unabsorbed dietary components by the gut microbiota that has been received more attention, recently due to their role in the gut barrier and metabolism^13,14^. Previously, some randomized controlled trials showed that a western-style diet promotes inflammation, changes the profile of gut microbiota to the obese pattern, and decreases the amount of beneficial gut microbiota, especially *Lactobacillus* sp. and *Bifidobacterium* sp^15,16^. However, the plant-based and Mediterranean diets increased the abundance of protective microbiota and the protectors of intestinal barrier including *Bifidobacteria* and *Lactobacillus*^17^. Moreover, butyrate-producing bacteria including increased and inflammation-inducing lipopolysaccharides decreased^17–19^. In our previous study, the gut *Actinobacteria* population significantly increased in women who received a low-carb diet, however the *Proteobacteria* population significantly decreased in this group compared to the habitual diet*.* Moreover, changes in the gut microbiota population affected on the serum atherogenic and antioxidant status^20^. Following this, we hypothesized that the metabolites of the gut microbiota, especially SCFAs, will change after alterations in the microbiota composition. Acetate, propionate, and butyrate are the main SCFAs with 60:20:20 molar ratios in the colon and stool, depending on the dietary components and the diversity of gut microbiota^21,22^. In a recent cell culture study, a potential correlation was founded between fecal SCFAs and inflammation^23^; however this association has not been studied in a human population, up to date. Herein, we compared the effects of the low-carb (LCD) and habitual (HD) diets on fecal level of SCFAs, and some inflammatory markers in women who participated in our previous study^20^. In addition, the correlation between serum inflammatory markers and fecal SCFAs was assessed.

## Results

**Figure 1:**
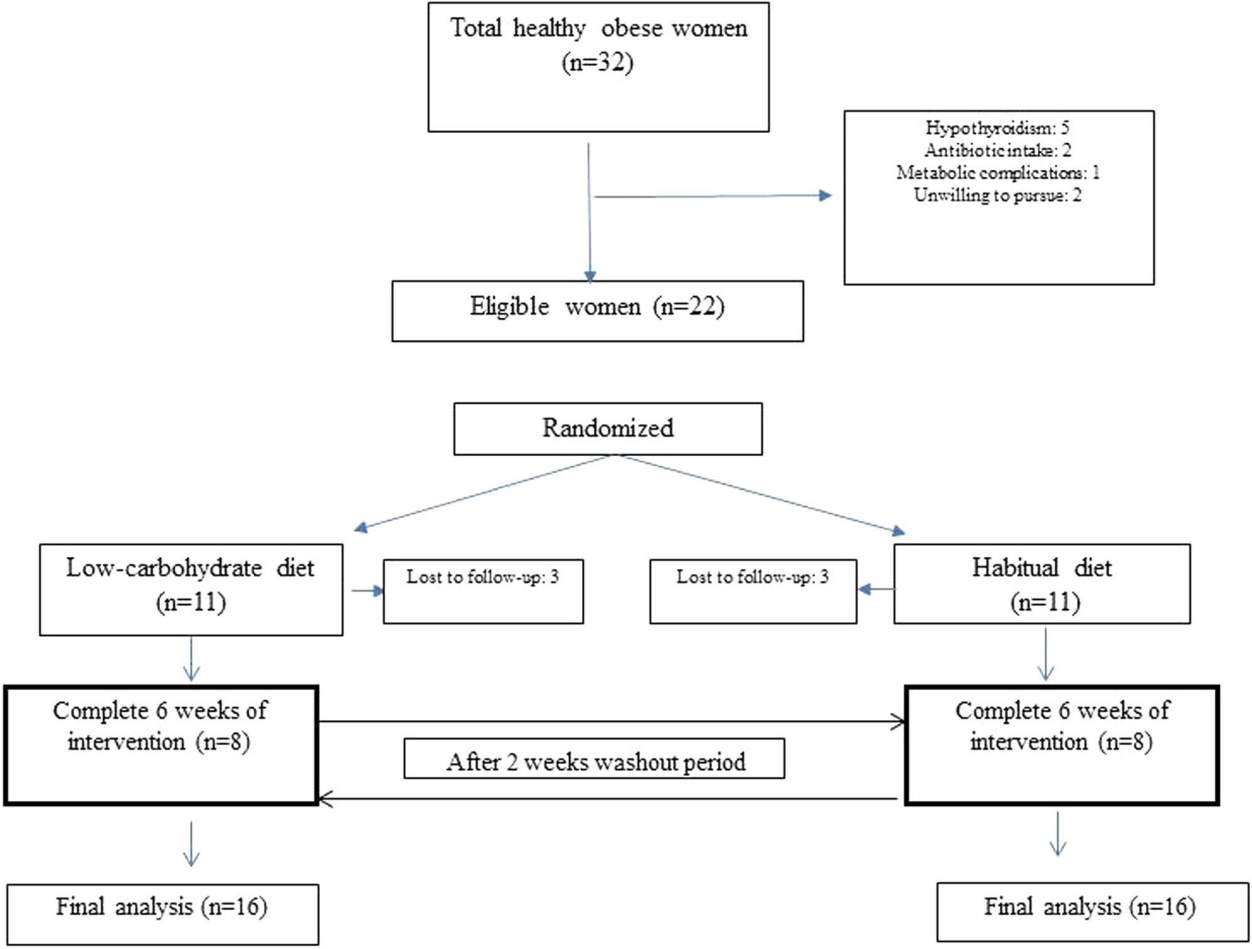
The study protocol.

The study protocol is illustrated in Fig. 1. Total calorie, protein, fat, carbohydrate, and fiber had no significant difference between the two studied groups at the baseline^20^.

As shown in Table 1, a significant difference was seen in weight and waist circumference (WC) at the end of intervention in both dietary groups (*p* < 0.001 in all). The mean changes in weight and WC were not statistically significant between the two groups. Waist-to-hip ratio (WHR) significantly decreased from baseline up to the end in the LCD (*p* = 0.001) and HD (*p* = 0.01) groups. Serum insulin and HOMA-IR significantly decreased (*p* = 0.001 and *p* = 0.003, respectively) in the HD compared to the LCD. Serum interleukin-6 (IL-6) significantly increased in the HD group from baseline up to the end (*p* < 0.001). Serum high sensitive C-reactive protein (hs-CRP) level significantly decreased in both dietary groups at the end (*p* = 0.01 in the LCD and *p* = 0.04 in the HD). There was no significant difference between the two dietary groups in *Bacteroidetes* and *Firmicutes* population, before and after the intervention. Positive-*Actinobacteria* and *Proteobacteria* participants significantly increased and decreased after six weeks of the LCD intake (*p* = 0.002 and *p* = 0.004, respectively). Positive-*Actinobacteria* participants were significantly higher in the LCD than the HD group at the end (*p* = 0.03). Fecal acetic acid significantly increased after six weeks of intervention in both dietary groups (*p* < 0.001), however the fecal acetic acid level was significantly higher in the LCD compared to the HD group at the end (*p* < 0.001). Fecal propionic acid significantly increased in both dietary groups from baseline up to the end (*p* < 0.001). After six weeks of intervention, propionic acid level was significantly higher in the stool of participants received the LCD than the HD (*p* = 0.003). Butyric acid significantly increased in both dietary groups at the end, however, this elevation was significantly higher in the stool of women received the LCD than the HD (*p* < 0.001 in all comparisons). As shown in Table 2, the mean change of WHR was significantly higher in the LCD than in the HD group (*p* = 0.01). The decrease in IL-6 was significantly higher in the serum of women received the LCD than the HD (*p* = 0.009). The mean changes of serum insulin and HOMA-IR were significantly higher in women on a HD than the LCD (*p* = 0.008 and *p* = 0.01, respectively). The mean changes of fecal acetic, propionic, and butyric acid were significantly higher in women on the LCD than the HD (*p* < 0.001, *p* = 0.001 and* p* < 0.001, respectively).

**Table 1:** Anthropometric, glucose metabolism and inflammatory markers.

Variables	Groups		*p* Value†
	LCD (n = 16)	HD (n = 16)	
Weight, kg			
Before	84.97 ± 2.4	85.27 ± 2.49	0.93
After	81.1 ± 2.5	81.01 ± 2.53	0.98
*p* Value	** < 0.001**	** < 0.001**	
Waist circumference, cm			
Before	111.1 ± 1.7	111.9 ± 2.03	0.78
After	108.5 ± 1.7	110.08 ± 1.8	0.5
*p* Value	** < 0.001**	** < 0.001**	
WHR			
Before	0.94 ± 0.01	0.91 ± 0.01	0.2
After	0.88 ± 0.01	0.90 ± 0.01	0.9
*p* Value	**0.001**	**0.01**	
FBS, mg/dl			
Before	86.1 ± 3.3	87.4 ± 1.7	0.7
After	81.2 ± 2.6	85.9 ± 2.4	0.2
p value	0.36	**0.01**	
Insulin, IU/ml			
Before	11.4 ± 1.4	13.5 ± 2.3	0.4
After	12.04 ± 1.8	8.2 ± 1.5	0.12
*p* Value	0.6	**0.001**	
HOMA-IR			
Before	2.47 ± 0.31	2.98 ± 0.57	0.42
After	2.55 ± 0.38	1.67 ± 0.33	0.09
*p* Value	0.82	**0.003**	
IL-6, pg/ml			
Before	3.31 ± 0.7	1.83 ± 0.22	0.06
After	1.7 ± 0.8	2.06 ± 0.55	0.62
*p* Value	0.69	** < 0.001**	
hs-CRP, mg/ L			
Before	4.17 ± 0.8	3.7 ± 0.72	0.68
After	2.42 ± 0.43	2.86 ± 0.59	0.56
*p* Value	0.01	0.04	
Acetic acid, mmol/L			
Before	30.15 ± 0.44	30.18 ± 0.45	0.95
After	41.57 ± 0.61	34.34 ± 0.79	** < 0.001**
*p* Value	** < 0.001**	** < 0.001**	
Propionic acid, mmol/L			
Before	8.37 ±0.24	8.47 ± 0.28	0.48
After	12.54 ± 0.48	10.34 ± 0.47	**0.003**
*p* Value	** < 0.001**	** < 0.001**	
Butyric acid, mmol/L			
Before	7.69 ± 0.21	7.81 ± 0.2	0.7
After	13.27 ± 0.24	9.5 ± 0.29	< **0.001**
*p* Value	** < 0.001**	** < 0.001**	
Bacteroidetes			
Before	P: 15	P: 26	0.9
	N: 2	N: 0	
After	P: 16	P: 16	0.9
	N: 0	N: 0	
*p* Value	0.9	0.9	
Firmicutes			
Before	P: 16	P: 16	0.9
	N: 0	N: 0	
After	P: 16	P: 16	0.9
	N: 0	N: 0	
*p* Value	0.9	0.9	
Actinobacteria			
Before	P: 2	P: 2	0.9
	N: 14	N: 14	
After	P: 10	P: 3	0.03
	N: 6	N: 13	
*p* Value	**0.002**	0.3	
Proteobacteria			
Before	P: 14	P: 14	0.9
	N: 2	N: 2	
After	P: 8	P: 13	0.06
	N: 8	N: 3	
p value	**0.004**	0.5	

Data are expressed as means ± SD. The bold values are significant.

*LCD* low-carbohydrate diet; *HD* habitual diet; *WHR* waist to hip ratio; *FBS* fasting blood sugar; *HOMA-IR* homeostasis model assessment of insulin resistance. *IL-6* interleukin-6; *hs-CRP* high sensitive C-reactive protein; *P* positive; *N* negative.

^†^Differences between the groups were evaluated by the parallel repeated measures.

**Table 2:** Mean changes of the studied variables from baseline up to the end.

Variables	Groups		*p* Value
	LCD (n = 16)	HD (n = 16)	
Weight (kg)	− 4.3 ± 0.38	− 3.88 ± 0.44	0.52
Waist circumference (cm)	− 1.8 ± 0.3	− 2.6 ± 0.3	0.1
WHR	− 0.04 ± 0.01	− 0.01 ± 0.004	**0.01**
FBS mg/dl	− 1.4 ± 1.5	− 4.8 ± 1.8	0.16
Insulin IU/ml	0.63 ± 1.5	− 5.3 ± 1.3	**0.008**
HOMA IR	0.078 ± 0.35	− 1.3 ± 0.37	**0.01**
IL-6 pg/ml	− 1.6 ± 0.34	0.24 ± 0.58	**0.009**
hs-CRP mg/L	− 0.87 ± 0.41	− 1.7 ± 0.61	0.23
Butyric Acid mmol/L	5.57 ± 0.22	1.7 ± 0.2	** < 0.001**
Propionic Acid mmol/L	3.81 ± 0.31	1.87 ± 0.45	**0.001**
Acetic Acid mmol/L	11.4 ± 0.42	4.15 ± 0.8	** < 0.001**

Data are expressed as means ± SD; Differences between the groups were evaluated by the parallel repeated measures. The bold values are significant.

*LCD* low-carbohydrate diet; *HD* habitual diet; *WHR* waist to hip ratio; *FBS* fasting blood sugar; *HOMA-IR* homeostasis model assessment of insulin resistance. *IL-6* interleukin-6; *hs-CRP* high sensitive C-reactive protein.

Baseline comparisons between the LCD to HD and HD to LCD groups showed a significant difference in serum IL-6 and hs-CRP level (*p* = 0.02, and *p* < 0.001, respectively). Weight (*p* < 0.001), WC (*p* < 0.001), WHR (*p* = 0.04), serum fasting blood sugar (FBS) (p = 0.03), insulin (p = 0.001) and HOMA-IR (p = 0.003) significantly decreased in the HD to LCD group, after six weeks of intervention. In the LCD to HD group, weight (*p* < 0.001), waist circumference (*p* = 0.001), WHR (*p* = 0.01), serum IL-6 (*p* = 0.001) and hs-CRP (*p* = 0.009) significantly decreased after six weeks. Positive-*Actinobacteria* participants significantly increased in women on a HD to LCD at the end (*p* = 0.02) (Table 3). The mean changes of serum insulin and HOMA-IR were significantly higher in the HD to LCD than the LCD to HD group (*p* = 0.04, and *p* = 0.04, respectively). The mean changes of serum hs-CRP were significantly higher in the LCD to HD than in the HD to LCD group (*p* = 0.04). Other parameters had no significant difference between the two groups. (Table 4).

**Table 3:** Anthropometric, glucose metabolism and inflammatory markers.

Variable	Group		*p* Value^†^
	LCD to HD (n = 8)	HD to LCD (n = 8)	
Weight, kg			
Before	89.2 ± 3.4	84.7 ± 1.5	0.24
After	85.7 ± 3.3	80.2 ± 1.5	0.14
*p* Value^‡^	** < 0.001**	** < 0.001**	
Waist circumference, cm			
Before	105.1 ± 2.9	107.7 ± 1.1	0.1
After	99.7 ± 2.8	97.1 ± 0.8	0.37
*p* Value	**0.001**	** < 0.001**	
WHR			
Before	0.92 ± 0.02	0.91 ± 0.01	0.5
After	0.89 ± 0.02	0.9 ± 0.01	0.7
*p* Value	**0.01**	**0.04**	
FBS, mg/dl			
Before	87.3 ± 3.8	87.3 ± 2.1	0.9
After	84.6 ± 3.9	83.1 ± 1.7	0.7
*p* Value	0.2	**0.03**	
nsulin, IU/ml			
Before	13.2 ± 2.3	13.6 ± 1.9	0.9
After	11.8 ± 2.6	8.1 ± 1.08	0.1
*p* Value	0.3	**0.001**	
HOMA-IR			
Before	2.89 ± 0.5	3 ± 0.49	0.8
After	2.49 ± 0.5	1.7 ± .23	0.17
*p* Value	0.27	**0.003**	
IL-6 pg/ml			
Before	2.7 ± .27	1.45 ± 0.14	< 0.001
After	1.85 ± 0.25	1.8 ± 0.65	0.9
*p* Value	**0.001**	0.61	
hs-CRP mg/L			
Before	5.5 ± 1.08	2.8 ± 0.4	0.02
After	3.4 ± 0.76	2.15 ± 0.35	0.15
*p* Value	**0.009**	0.13	
Butyric Acid mmol/L			
Before	8 ± 0.21	7.69 ± 0.19	0.2
After	11.56 ± 0.64	10.8 ± 0.57	0.38
*p* Value	** < 0.001**	** < 0.001**	
Propionic Acid mmol/L			
Before	8.99 ± 0.31	8.5 ± .23	0.2
After	12.3 ± 0.66	11.1 ± 0.59	0.18
*p* Value	** < 0.001**	** < 0.001**	
Acetic Acid mmol/L			
Before	30.8 ± 0.34	29.7 ± 0.55	0.1
After	37.95 ± 1.2	37.1 ± 1.4	0.6
*p* Value	** < 0.001**	** < 0.001**	
*Bacteroidetes*			
Before	P: 8	P: 7	0.9
	N: 0	N: 1	
After	P: 8	P: 8	0.9
	N: 0	N: 0	
*p* Value	0.9	0.9	
*Firmicutes*			
Before	P: 8	P: 8	0.9
	N: 0	N: 0	
After	P: 8	P: 8	0.9
	N: 0	N: 0	
*p* Value	0.9	0.9	
*Actinobacteria*			
Before	P: 1	P: 1	0.9
	N: 7	N: 7	
After	P: 3	P: 4	0.08
	N: 5	N: 4	
*p* Value	0.08	**0.001**	
*Proteobacteria*			
Before	P: 8	P: 6	0.9
	N: 0	N: 2	
After	P: 6	P: 6	0.06
	N: 2	N: 2	
*p* Value	0.08	0.58	

The bold values are significant.

*LCD to HD* low-carbohydrate diet to habitual diet; *HD to LCD* habitual diet to low-carbohydrate diet; each diet for six weeks with two weeks washout period; *WHR* waist to hip ratio; *FBS* fasting blood sugar; *HOMA-IR* homeostasis model assessment of insulin resistance. *IL-6* interleukin-6; *hs-CRP* high sensitive C-reactive protein; *P* positive; *N* negative.

^†^Differences between the groups were evaluated by the parallel repeated measures.

**Table 4:** Mean changes of variables in the intervention groups.

Variables	Group		*p* Value
	LCD to HD (n = 8)	HD to LCD (n = 8)	
Weight, kg	− 3.4 ± 0.43	− 4.3 ± 44	0.1
Waist circumference, cm	− 5.3 ± 1.3	− 3.6 ± 0.74	0.2
WHR	− 0.0 ± 0.01	− 0.01 ± 0.005	0.1
FBS, mg/dl	− 2.7 ± 2.09	− 4.1 ± 1.8	0.6
Insulin IU/ml	− 1.4 ± 1.4	− 5.5 ± 1.3	**0.04**
HOMA IR	− 0.4 ± 0.3	− 1.3 ± 0.3	**0.04**
IL-6 pg/ml	− 0.86 ± 0.2	0.35 ± .66	0.09
hs-CRP mg/L	− 2.1 ± 0.69	− 0.62 ± 0.38	**0.04**
Butyric acid mmol/L	3.5 ± 0.6	3.1 ± 0.5	0.6
Propionic acid mmol/L	3.3 ± 0.56	2.6 ± 0.47	0.3
Acetic acid mmol/L	7.2 ± 1.2	7.4 ± 1.2	0.9

The bold values are significant.

*LCD to HD* low-carbohydrate diet to habitual diet; *HD to LCD* habitual diet to low-carbohydrate diet; each diet for six weeks with two weeks washout period; *WHR* waist to hip ratio; *FBS* fasting blood sugar; *HOMA-IR* homeostasis model assessment of insulin resistance. *IL-6* interleukin-6; *hs-CRP* high sensitive C-reactive protein.

The mean changes of fecal SCFAs were adjusted for the baseline parameters and no significant effect was observed. Adjusted for the baseline parameters, fecal level of butyric, propionic, and acetic acid were significantly different between women on the LCD and HD (*p* < 0.001, *p* = 0.02, and *p* < 0.001, respectively). Only serum insulin level showed a significant effect on the fecal level of propionic acid (*p* = 0.04). Increase in serum insulin level decreased fecal level of propionic acid by 5.3-folds (95% CI =  − 2.7,  − 0.15). (Table 5).

**Table 5:** Effects of baseline parameters on fecal short-chain fatty acids in the groups^†^.

Variables	Beta ± SE	OR	95%CI	p value
Butyric acid				
Group	− 3.7 ± 0.5	− 0.88	− 4.79, − 2.67	** < 0.001**
Age	− 0.04 ± 03	− 0.12	− 0.11, 0.04	0.3
Weight	− 0.04 ± 03	− 0.17	− 0.11, 0.04	0.3
Waist circumference	0.02 ± 0.05	0.09	− 0.07, 0.12	0.6
WHR	− 1.8 ± 4.9	− 0.06	− 12, 8.35	0.7
FBS	0.03 ± 0.08	0.15	− 0.14,0.2	0.7
Insulin	0.05 ± 0.4	0.2	− 0.76 ± 0.86	0.8
hs-CRP	− 0.01 ± 0.09	− 0.02	− 0.2, 0.17	0.9
IL-6	0.03 ± 0.1	0.03	− 0.18, 0.24	0.76
HOMA-IR	− 0.21 ± .8	− 0.17	− 3.9, 3.6	0.9
Acetic acid	− 0.12 ± 0.1	− 0.1	− 0.34, 0.09	0.2
Propionic acid	0.008 ± 0.22	0.004	− 0.45, 0.46	0.97
Propionic acid				
Group	− 2.27 ± 0.89	− 0.63	− 4.2, − 0.42	**0.02**
Age	− 0.02 ± 0.06	− 0.07	− 0.15, 0.11	0.7
Weight	− 0.08 ± 0.06	0.46	− 0.04, 0.2	0.2
Waist circumference	− 0.03 ± 0.08	− 0.14	− 0.2, 0.1	0.7
WHR	9 ± 8.5	0.35	− 8.8, 26.8	0.3
FBS	− 0.2 ± .15	− 1.2	− 0.5, 0.09	0.16
Insulin	− 1.3 ± 0.7	− 5.3	− 2.7, − 0.15	**0.04**
hs-CRP	− 0.23 ± 0.15	− 0.4	− 0.55, 0.08	0.14
IL-6	− 0.03 ± 0.2	− 0.03	− 0.4,0.35	0.9
HOMA-IR	5.8 ± 3.17	5.8	− 0.8, 12.4	0.08
Acetic acid	0.17 ± 0.18	0.17	− 0.2, 0.6	0.3
Butyric acid	0.54 ± 0.47	0.2	− 0.44, 1.5	0.3
Acetic acid				
Group	− 8.8 ± 1.2	− 1	− 11.4, − 6.2	** < 0.001**
Age	0.001 ± 0.08	0.001	− 0.18, 0.18	0.9
Weight	0.06 ± 0.08	0.14	− 0.12, 0.24	0.47
Waist circumference	− 0.007 ± 0.1	− 0.06	− 0.24, 0.23	0.9
WHR	2.6 ± 11.8	0.04	− 22.1, 27.4	0.8
FBS	− 0.37 ± 0.2	− 0.87	− 0.8, 0.05	0.08
Insulin	− 1.2 ± 0.95	− 2.1	− 3.2, 0.7	0.2
hs-CRP	− 0.08 ± 0.2	− 0.06	− 0.5, 0.35	0.7
IL-6	− 0.3 ± 0.25	− 1.4	− 0.87,0.17	0.17
HOMA-IR	6.4 ± 4.4	2.6	− 2.7, 15.65	0.16
Propionic acid	− 0.6 ± 0.54	− 0.15	− 1.7, 0.45	0.26
Butyric acid	− 0.92 ± 0.66	− 0.2	− 2.3, 0.45	0.2

Regression analysis was performed by adjusting the baseline parameters; groups: low fat and low carbohydrate. The bold values are significant.

*WHR* waist to hip ratio; *FBS* fasting blood sugar; *HOMA-IR* homeostasis model assessment of insulin resistance; *IL-6* interleukin-6; *hs-CRP* high sensitive C-reactive protein.

^†^Group: low carbohydrate (LCD) versus habitual (HD) diet.

Moreover, fecal butyric, propionic, and acetic acid had no significant difference between the LCD to HD and the HD to LCD group, adjusting for the parameters. Serum hs-CRP showed a significant effect on fecal level of butyric acid (*p* = 0.04). Increase in serum hs-CRP decreased the percentage of fecal butyric acid by 25%. Fecal propionic acid showed a significant effect on butyric acid level (*p* = 0.03). Serum FBS and insulin showed a significant effect on fecal level of acetic acid (*p* = 0.009 and *p* = 0.01, respectively). Elevated serum FBS and insulin increased fecal level of acetic acid by 2.8 and 8.9-folds (95% CI = 0.34, 1.9 and 1.2, 9.2), respectively. Fecal propionic and butyric acid showed a significant effect on acetic acid (*p* = 0.01 and *p* = 0.02, respectively). (Table 6).

**Table 6:** Effects of baseline parameters on fecal short-chain fatty acids in the groups^†^.

Variables	Beta ± SE	OR	95%CI	*p* Value
Butyric acid				
Group^†^	0.45 ± 1.1	0.12	− 1.9, 2.9	0.67
Age	0.02 ± 0.07	0.08	− 0.12, 0.17	0.7
Weight	0.02 ± 0.12	0.09	− 0.23, 0.27	0.87
Waist circumference	0.07 ± 0.17	0.27	− 0.29, 0.42	0.67
WHR	11.58 ± 14.9	0.38	− 20.2, 43.4	0.45
FBS	0.33 ± 0.16	1.8	− 0.002, 0.67	0.05
Insulin	0.05 ± 0.4	0.2	− 0.76 ± 0.86	0.8
hs-CRP	− 0.47 ± 0.2	− 0.75	− 0.9, − 0.02	**0.04**
IL-6	1.07 ± 0.63	0.5	− 0.27, 2.4	0.1
HOMA-IR	− 5.1 ± 3.6	− 4.6	− 12.9, 2.7	0.2
Acetic acid	− 0.06 ± 0.2	− 0.05	− 0.6, 0.48	0.8
Propionic acid	1.1 ± 0.47	0.56	0.12, 2.1	**0.03**
Propionic acid				
Group	− 1.6 ± 1.3	− 0.43	− 4.36, 1.04	0.2
Age	0.03 ± 0.08	0.12	− 0.15, 0.22	0.7
Weight	0.08 ± 0.13	0.4	− 0.2, 0.35	0.6
Waist circumference	0.006 ± 0.18	0.02	− 0.38, 0.39	0.9
WHR	21.2 ± 16.1	0.73	− 13.4, 55.7	0.2
FBS	0.18 ± 0.19	1.07	− 0.2, 0.6	0.3
Insulin	0.34 ± 0.98	1.36	− 1.7, 2.4	0.7
hs-CRP	− 0.39 ± 0.26	− 0.65	− 0.94, 0.16	0.15
IL-6	− 0.53 ± 0.79	− 0.27	− 2.2,1.2	0.5
HOMA-IR	− 1.6 ± 4.5	− 1.5	− 11.3, 8.1	0.7
Acetic acid	− 0.009 ± 0.3	− 0.008	− 0.62, 0.61	0.9
Butyric acid	− 0.34 ± 0.84	− 0.13	− 2.1, 1.5	0.7
Acetic acid				
Group	− 2.3 ± 2.4	− 0.26	− 7.5, 2.8	0.35
Age	0.17 ± 0.16	0.27	− 0.17, 0.52	0.3
Weight	0.03 ± 0.24	0.06	− 0.49, 0.55	0.9
Waist circumference	0.25 ± 0.34	0.5	− 0.48, 0.99	0.5
WHR	13.2 ± 30.7	0.2	− 52.7, 79.1	0.67
FBS	1.1 ± 0.37	2.8	0.34, 1.9	**0.009**
Insulin	5.2 ± 1.9	8.9	1.2, 9.2	**0.01**
hs-CRP	− 0.08 ± 0.5	− 0.06	− 1.1, 0.96	0.87
IL-6	− 2.4 ± 1.5	− 0.5	− 5.6,0.8	0.13
HOMA-IR	− 2.3 ± 2.4	− 0.26	− 7.5, 2.8	0.35
Propionic acid	2.8 ± 0.99	0.64	0.7, 4.9	**0.01**
Butyric acid	− 4. 2 ± 1.6	− 0.69	− 7.6, − 0.75	**0.02**

Regression analysis was performed by adjusting the baseline parameters; groups: low carbohydrate to habitual diet and habitual to low carbohydrate diet. The bold values are significant.

*WHR* waist to hip ratio; *FBS* fasting blood sugar; *HOMA-IR* homeostasis model assessment of insulin resistance. *IL-6* interleukin-6; *hs-CRP* high sensitive C-reactive protein.

^†^Group: low-carbohydrate to habitual diet (LCD to HD) versus habitual to low-carbohydrate diet (HD to LCD) group.

In the LCD group, fecal level of propionic acid showed a significant correlation with the positive- *Actinobacteria* population (r = 0.45, *p* = 0.04). Moreover, a significant correlation was shown between changes in fecal level of propionic acid with serum IL-6 (r = 0.46, *p* = 0.04). In the HD to LCD group, a significant correlation was founded between the positive- *Proteobacteria* population and fecal level of propionic acid (r = 0.7, *p* = 0.005). (Table 7).

**Table 7:** Correlation analysis between the gut phylum and inflammatory markers with fecal short-chain fatty acids in the studied groups.

Grouping	Variables	Acetic acid	Propionic acid	Butyric acid
LCD	*Actinobacteria*	r = − 0.16; *p* = 0.5	**r = 0.45; *p* = 0.04**	r = − 0.02; *p* = 0.9
	*Proteobacteria*	r = 0.19; *p* = 0.4	r = − 0.14; *p* = 0.6	r = − 0.27; *p* = 0.27
	hs-CRP	r = 0.29; *p* = 0.2	r = 0.42; *p* = 0.08	r = 0.02; *p* = 0.9
	IL-6	r = 0.02; *p* = 0.9	**r = 0.43; *p* = 0.01**	r = − 0.27; *p* = 0.2
HD	*Actinobacteria*	r = 0.38; *p* = 0.14	r = 0.49; *p* = 0.05	r = − 0.04; *p* = 0.9
	*Proteobacteria*	r = − 0.07; *p* = 0.8	r = 0.42; *p* = 0.1	r = 0.05; *p* = 0.8
	hs-CRP	r = − 0.09; *p* = 0.7	r = 0.08; *p* = 0.7	r = 0.06; *p* = 0.8
	IL-6	r = 0.02; *p* = 0.9	r = 0.08; *p* = 0.7	r = 0.06; *p* = 0.8
LCD to HD	*Actinobacteria*	r = − 0.07; p = 0.8	r = − 0.07; p = 0.8	r = − 0.07; p = 0.8
	*Proteobacteria*	r = 0.36; *p* = 0.2	r = 0.13; *p* = 0.65	r = − 0.05; *p* = 0.8
	hs-CRP	r = 0.3; *p* = 0.2	r = 0.4; *p* = 0.15	r = 0.37; *p* = 0.18
	IL-6	r = − 0.09; *p* = 0.7	r = 0.04; *p* = 0.9	r = − 0.3; *p* = 0.3
HD to LCD	*Actinobacteria*	r = − 0.3; *p* = 0.3	r = − 0.15; *p* = 0.6	r = − 0.08; *p* = 0.7
	*Proteobacteria*	r = 0.08; *p* = 0.78	**r = 0.6; *p* = 0.02**	r = 0.25; *p* = 0.4
	hs-CRP	r = 0.06; *p* = 0.8	r = 0.36; *p* = 0.2	r = − 0.12; *p* = 0.7
	IL-6	r = − 0.3; *p* = 0.25	r = − 0.06; *p* = 0.8	r = − 0.3; *p* = 0.27

Correlation analysis was performed by the Kendall’s tau-b and Spearman test. The bold values are significant.

*LCD* low-carbohydrate diet; *HD* habitual diet; *LCD to HD* low-carbohydrate diet to habitual diet; *HD to LCD* habitual diet to low-carbohydrate diet; each diet for six weeks with two weeks washout period; *IL-6* interleukin-6; *hs-CRP* high sensitive C-reactive protein.

## Discussion

As a novel finding, serum hs-CRP level showed a significant effect on fecal level of butyric acid. Moreover, a mutual relationship was observed between the *Actinobacteria* population and fecal level of propionic acid. Moreover, a mutual relationship was observed between fecal level of propionic acid and serum IL-6, as an initiating factor of inflammatory pathways. The SCFAs, carboxylic acids with aliphatic tails of 1–6 carbons, are volatile bacterial metabolites of unabsorbed/ undigested food components, especially carbohydrates in the large intestine. Non-digestible dietary fibers are the main substrates for bacterial fermentation to produce acetic (C2), propionic (C3), and butyric (C4) acids, as the most abundant SCFAs in the colon which have various impacts on human metabolism and health^24^. Different microorganisms in the gut produce SCFAs through various pathways^25–28^. The main butyrate producing-bacteria in the human gut belong to the *Firmicutes* phyla. Moreover, sugar and lactate-utilizing bacteria, such as *Eubacterium hallii* and *Anaerostipes* *spp*. produce butyrate from lactate and acetate^29^. The *Actinobacteria, Bacteroidetes, Fusobacteria, Proteobacteria, Spirochaetes*, and *Thermotogae* can produce butyrate through an increase in gene expression of butyryl-CoA dehydrogenase, butyryl-CoA transferase and/or butyrate kinase^30^. The *Actinobacteria* phyla regulate the production of acetate and propionate in the gut^31^. These data are consistent with our study that the positive-*Actinobacteria* women increased, but positive-*Proteobacteria* population decreased in the LCD group^20,32^. In the present study, the *Actinobacteria* showed a significant correlation with fecal level of propionic acid. Alteration in fecal SCFAs occurred due to changes in phyla population in the gut^33^. Obesity has been associated with increase in the *Firmicutes* and decrease in the *Bacteroidetes* population in previous studies^34,35^. In the present study, no change in the *Firmicutes* and *Bacteroidetes* population was observed before and after intervention. Difference in foods, region, culture, climate, and ethnicity make variations in these results that create novelty in this field.

The SCFAs induce epigenetic modifications such as changes in DNA methylation and micro-RNA expression^36^. They regulate appetite, lipogenesis, gluconeogenesis, and inflammation which have potential effects on health status, susceptibility to obesity, and related complications^37^. The SCFAs affect inflammatory pathways via several mechanisms including regulating the cytokine production, activating the acetylation of G-protein-coupled receptors (GPCRs), and tight junction proteins that finally strengthen the intestinal integrity, which is one of the important factors for inflammatory pathways^38^. There is a relationship between oxidative stress, inflammation, and the gut barrier status. Oxidative stress degrades the intestinal integrity by activating the signaling pathways of nuclear factor kappa- B (NF-κB), insulin receptor kinase, and mitogen-activated protein kinase (MAPK). Inflammation and damage to the intestinal barrier interact by regulating the expression of tumor necrosis factor (TNF), claudin-2, occludin, and zonula occludens-1 (ZO1). Oxidative stress directly promotes inflammation by inhibiting the NF-κB activity and the expression of TNF-α and interleukin-1 beta (IL-1β)^39^. Our previous study showed that the LCD increases the *Actinobacteria* population in the gut and improves serum total antioxidant capacity which is associated with higher capability of the body for reactive oxygen scavenges. Moreover, decrease in the *Proteobacteria* population lead to lower oxidant status in the body^19^. Association between the SCFAs and serum inflammatory markers has been studied in some previous animal models^40,41^. For example, dietary sodium butyrate supplementation reduced serum IL-6 and TNF-α level in pigs. The number of *Clostridium* and *Escherichia coli* decreased, but the number of *Lactobacillus* *spp* increased in the gut of pigs^40^. *Lactobacillus* is facultative anaerobic bacteria belong to the *Firmicutes* phyla that metabolize carbohydrates to produce lactic acid^41^. We did not assess the species of bacteria in each phylum in the present study. Participants only studied for positive or negative phyla and no difference was observed in the *Firmicutes* population between the groups before and after six weeks.

Dietary composition changes the gut microbiota and the produced SCFAs, as the final metabolites of undigested food in the large intestine^42,43^. Previous human study reported that a western style diet with a high intake of refined carbohydrates and saturated fats promotes inflammation by a change in the *Actinobacteria* population^16^. But, plant-based diet increased butyrate-producing bacteria belonging to the *Actinobacteria* phyla, however decreased inflammation-inducing bacteria, as the members of *Proteobacteria* phyla^17–19^. Our results are in accordance with the mentioned studies. Dietary fat was provided from PUFAs in the present LCD that leads to increase in *Actinobacteria* population in the gut. Higher *Actinobacteria* population correlated with higher fecal level of propionate and lower serum IL-6. Animal models feeding propionate and butyrate-enriched high-fat diet were resistant to obesity and improved blood glucose levels^44–47^. In human studies, propionate supplementation increased the satiety hormones including peptide YY (PYY) and glucagon-like peptide (GLP-1), which have been related to lower serum FBS and higher insulin secrection in the body^48,49^. Herein, fecal level of butyric acid significantly increased in the LCD compared to the HD group. As we previously reported, positive- *Actinobacteria* and *Proteobacteria* participants significantly increased and decreased after the LCD, respectively^20^. An inverse association has been reported between the intestinal propionate and butyrate level with inflammation^50^ which is consistent with our results. Propionate inhibits histone deacetylases (HDACs) and activates histone acetyltransferases (HATs), which are associated with inflammatory- and immune-regulatory pathways^51^. In addition, it regulates cytokine expression in T-cells and generates the regulatory T-cells (Tregs) through HDAC inhibition^52^. Recently, a population-based study in China showed a positive correlation between the butyrate and BMI status. No statistical significant difference was observed between the SCFAs-producers of bacteria and BMI. Plasma levels of SCFAs positively associated with BMI. They concluded that the colonic fermentation of undigested/unabsorbed foods differs in adults with and without overweight/obesity^53^. Our results showed no correlation between the fecal levels of butyrate with anthropometric measures, and serum inflammatory markers. Differences in the ethnicity make variety in the gut microbiota population and their species that change the final produced metabolites, especially SCFAs. A recent study on morbid obese patients referred for one anastomosis gastric bypass- mini gastric bypass showed the beneficial effect of probiotic on serum IL-6, TNF-α and hs-CRP after 16 weeks of supplementation, however the mean changes of serum TNF-α was only statistically significant between the supplemented [-6.18 (-12.69, 0.32)] and placebo [4.04 (− 1.18, 9.26)] groups. Moreover, serum FBS, insulin and HOMA-IR improved at the end of study in the supplemented group, but the mean changes were not statistically significant between the two groups. In addition anthropometric measures including the percentage excess weight loss, WC, BMI and weight significantly decreased after sixteen weeks of supplementation in the probiotic group, however the mean changes of WC was not statistically significant between the supplemented and placebo group^54^. Our results are in accordance with the mentioned study about inflammatory markers; however we did not measure serum TNF-α. This study was a randomized controlled trial that compared the effect of probiotic supplementation containing seven species of bacteria belonging to the *Actinobacteria* and *Firmicutes* phyla with placebo on anthropometric measures, glycemic indices and serum inflammatory markers in patients under the bypass surgery that is different in the study design and intervention with our study. But, the beneficial effect of the *Actinobacteria* phyla on serum inflammatory markers has been observed in both studies. Recent reviews have been discussed about the role of SCFAs in the redox signaling pathways, protection against bone loss, and inflammation^13,14^, however no human randomized controlled trial with a cross-over design was founded in this field. Therefore, similar to all novel studies, the present study has some limitations. The sample size was very small, and only women were enrolled. More clinical trials with larger sample sizes are needed. The levels of SCFAs were only measured in the stool and there is no data about their levels in serum. In addition, we did not study the absorption of SCFAs. It is still an open question whether the elevation of fecal SCFAs is because of a decrease in the gut absorption or not. The bacterial species did not assay in the present study. Determination of actual values of phyla and species make the changes more debatable. This is a new field of study that needs more future attempts to clearly describe underlying mechanisms and impacts of these changes in human health. A complex interaction between the genetic background, the gut microbiota, and diet has been opened a new target and tool for the personalized medical nutrition therapy.

## Materials and methods

### Participants and interventions

Block randomization was used as two groups with 5-number blocks, including four participants in each block. The randomization unit was the person, and we used random allocation software for this purpose. Random coded boxes were used for concealment. In this method, cans with similar weight, shape, and color, which were numbered according to the random sequence, was used. Our previous study on effects of the HD and LCD on the gut microbiota in women with obesity (BMI ≥ 30 kg/m^2^)^20^, is followed here by measuring the diet’s impact on fecal levels of SCFAs, as the main metabolites of the gut phyla. The hypocaloric HD and LCD were prescribed for six weeks with two weeks of washout period. According to the previous study, two weeks is sufficient for removing the effect of diets on the gut microbiota^55^. Hypocalorie diets were prescribed with 500 kcal reductions from the total daily calorie requirements for 0.5 kg weight loss in each week. From total energy requirements, 55%, 25%, and 20% were provided from fat, protein, and carbohydrate, respectively. The HD was a 500 kcal- reduced calorie diet that provided 20%, 15%, and 65% of total daily calories from fat, protein, and carbohydrate. The PUFAs were advised as the main source of dietary fat and fiber was prescribed in similar amounts (20 g/day) in both diets. In the washout period, the weight maintenance HD was prescribed based on 1.4–1.5 × resting energy expenditure for all participants. Compliance was assessed by the food diary and participants who followed < 80% of the dietary plan were excluded. The present study was ethically approved by the ethical committee of Zanjan University of Medical Sciences (IR.ZUMS.REC.1400.094). The informed consent form was obtained from all subjects. The present trial has been registered at the IRCT on 08-01-2021 under the registration number: IRCT20200929048876N3. All of the procedure was performed according to the Declaration of Helsinki.

### Anthropometric and biochemical measurements

Anthropometric measurements were recorded, and fasting blood samples were collected in our previous study^20^. Fasting serum insulin, IL-6, and hs-CRP were measured according to the ELISA method based on the manufacturer’s instruction (Pars Azmoon Co., Iran). The HOMA-IR was computed according to the below formula;

$$ \frac{{fasting\;glucose\;\left( {\frac{{mg}}{{dl}}} \right) \times insulin\left( {\frac{{mU}}{L}} \right)}}{{405}}. $$

### Extraction of SCFAs

The stool sample was gathered at the baseline and end of each dietary intervention and maintained in a refrigerator ( − 80 °C) for final analysis. The fecal SCFA analysis was carried out using gas chromatography-mass spectrometry (GC–MS). Before GC analysis, the fecal samples were subjected to an acid–base treatment followed by ether extraction, and derivatization.

The concentrations of volatile fatty acids were determined using a gas chromatography system (Agilent Chromatography System, model 7890B), equipped with a capillary column according to the method described previously^56^.

Briefly, 1 mL of 25% metaphosphoric acid was mixed with 1 g of sample in a centrifuge tube and the mixture was frozen overnight. The samples were then thawed, mixed with 0.4 mL of 25% NaOH, and vortexed, followed by the addition of 0.64 mL of 0.3 mol L^−1^ oxalic acid. The samples were centrifuged for 20 min at 3000g at 4 °C. Then, 2 mL of the supernatant was transferred into a gas chromatography vial. Helium as the carrier gas was used at a constant flow rate of 1 ml min^−1^. The initial column oven temperature was 50 °C for 2 min and increased to 70 °C at a rate of 10 °C min^-1^. Then, the temperature was increased to 85 °C at a rate of 3 °C min^−1^, then increased to 110 °C at a rate of 5 °C min^−1^, and then increased at a rate of 30 °C min^−1^ to a final temperature of 290 °C, where it was held for 5 min. The temperatures of the front inlet, transfer line, and mass source were set at 260 °C, 290 °C, and 230 °C, respectively.

## Statistical analyses

The number of participants was calculated according to the previous study with the effects of dietary intervention on the gut microbiota to change in the production of SCFAs, as the post-hoc endpoint^57^. Considering a power of 80% in a two-sided test, and α = 0.05 (type I error), eight people were sufficient to show this effect. Therefore, eight participants were randomly selected from our previous study^20^. Correlation analysis was performed by the Kendall’s and Spearman tests. The effects of the dietary interventions on all outcomes were analyzed using SPSS 18v through parallel repeated measures. A linear regression model was used to adjust the effect of baseline variables on outcomes. Analysis was performed in two models of grouping; (1) the LCD *vs.* HD, and (2) the LCD to HD *vs.* HD to LCD.

### Ethical statement

All of the procedure was performed according to the Declaration of Helsinki. The protocol was approved by the ethical committee of Zanjan University of Medical Sciences, Zanjan, Iran (IR.ZUMS.REC.1400.094).

## Data Availability

The datasets used and/or analysed during the current study available from the corresponding author on reasonable request.
